# Feasible diet and circadian interventions reduce in vivo progression of FLT3‐ITD‐positive acute myeloid leukemia

**DOI:** 10.1002/cam4.6949

**Published:** 2024-02-09

**Authors:** Megan Rodriguez, Baharan Fekry, Brianna Murphy, Mary Figueroa, Tiewei Cheng, Margaret Raber, Lisa Wartenberg, Donna Bell, Lisa Triche, Karla Crawford, Huaxian Ma, Kendra Allton, Ruwaida Ahmed, Jaime Tran, Christine Ranieri, Marina Konopleva, Michelle Barton, Cesar Nunez, Kristin Eckel‐Mahan, Joya Chandra

**Affiliations:** ^1^ Department of Pediatrics‐Research The University of Texas MD Anderson Cancer Center Houston Texas USA; ^2^ Institute of Molecular Medicine McGovern Medical School at the University of Texas Health Science Center (UT Health) Houston Texas USA; ^3^ University of Texas MD Anderson Cancer Center UT Health Houston Graduate School of Biomedical Sciences Houston Texas USA; ^4^ School of Public Health, Division of Epidemiology, Human Genetics and Environmental Sciences University of Texas Houston Texas USA; ^5^ Department of Pediatrics Patient Care The University of Texas MD Anderson Cancer Center Houston Texas USA; ^6^ Bionutrition Research Core The University of Texas MD Anderson Cancer Center Houston Texas USA; ^7^ Section of Molecular Hematology and Therapy, Department of Leukemia The University of Texas MD Anderson Cancer Center Houston Texas USA; ^8^ Department of Epigenetics and Molecular Carcinogenesis The University of Texas MD Anderson Cancer Center Houston Texas USA

**Keywords:** acute myeloid leukemia, circadian rhythm, diet, time‐restricted feeding

## Abstract

**Background:**

Acute myeloid leukemia (AML) with an internal tandem duplication in the fms‐like tyrosine kinase receptor 3 gene (FLT3‐ITD) is associated with poor survival, and few studies have examined the impact of modifiable behaviors, such as nutrient quality and timing, in this subset of acute leukemia.

**Methods:**

The influence of diet composition (low‐sucrose and/or low‐fat diets) and timing of diet were tested in tandem with anthracycline treatment in orthotopic xenograft mouse models. A pilot clinical study to test receptivity of pediatric leukemia patients to macronutrient matched foods was conducted. A role for the circadian protein, BMAL1 (brain and muscle ARNT‐like 1), in effects of diet timing was studied by overexpression in FLT3‐ITD‐bearing AML cells.

**Results:**

Reduced tumor burden in FLT3‐ITD AML‐bearing mice was observed with interventions utilizing low‐sucrose and/or low‐fat diets, or time‐restricted feeding (TRF) compared to mice fed normal chow ad libitum. In a tasting study, macronutrient matched low‐sucrose and low‐fat meals were offered to pediatric acute leukemia patients who largely reported liking the meals. Expression of the circadian protein, BMAL1, was heightened with TRF and the low‐sucrose diet. BMAL1 overexpression and treatment with a pharmacological inducer of BMAL1 was cytotoxic to FLT3‐ITD AML cells.

**Conclusions:**

Mouse models for FLT3‐ITD AML show that diet composition and timing slows progression of FLT3‐ITD AML growth in vivo, potentially mediated by BMAL1. These interventions to enhance therapy efficacy show preliminary feasibility, as pediatric leukemia patients responded favorable to preparation of macronutrient matched meals.

## Graphical abstract

Low‐fat and low‐sucrose diets (positively received by pediatric patients) enhanced anthracycline efficacy compared to mice fed normal chow. Time‐restricted feeding slows tumor progression compared to mice fed ad libitum and correlates with increased BMAL1 expression.

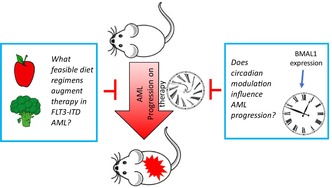

## 1 INTRODUCTION

In acute lymphocytic leukemia, the most frequently occurring pediatric blood cancer, diet modification resulting in weight loss has shown promise in improving treatment outcomes.^1^ However, the impact of diet modification on acute myeloid leukemia is less studied. Previous research found high‐fat diets and high‐fat‐diet‐induced obesity accelerate AML progression,^2^, ^3^ but these studies did not focus on any specific molecular subtype of AML. Point mutations in the fms‐like tyrosine kinase 3 gene and/or an internal tandem duplication (FLT3‐ITD) confer among the worst progression‐free survival rate (31%)^4^ for any hematological malignancy. Specific inhibition of the signaling pathways is ongoing with several kinase inhibitors having achieved approval; however, cost and access to these new agents can be challenging. In addition to targeting molecular features of AML to design improved treatments, diet composition and timing may influence therapy efficacy and have potential for global impact if studied in the context of broadly available chemotherapy for AML. In the United States, a significant percentage of children, including pediatric leukemia patients, consume nutritionally poor diets, high in both fat and added sugar.^5^, ^6^ Therefore, ensuring diet change is feasible and well‐received is a key aspect of a diet strategy for enhancing treatment outcomes.

Beyond diet composition, the impact of timing of diet and circadian regulation in AML is not well understood. A role for circadian regulation of AML stem cell activity has been documented using mice lacking brain and muscle ARNT‐like protein (BMAL1, also known as ARNTL/MOP3), or other core circadian proteins.^7^ However, the impact of diet modification or time‐restricted feeding (TRF), on leukemia progression or treatment efficacy has not been addressed. Furthermore, the ability of these behavioral modifications to modulate BMAL1 expression has not been studied in the context of FLT3‐ITD AML nor in treatment efficacy for the disease.

Here we utilized orthotopic mouse models of FLT3‐ITD AML and examined the impact of diet modification together with anthracycline treatment. Mouse diets were matched in macronutrient content to an observational study for pediatric leukemia patients in which receptivity to diet change was evaluated. We further assessed if diet timing, regardless of diet modification, can influence leukemia progression and examined BMAL1 protein expression and its pharmacological modulation in AML cell lines.

## 2 METHODS

### 2.1 Mouse studies

Four‐ to 5‐week‐old NOD‐SCID or NSG mice were obtained from the MD Anderson Cancer Center (MDACC) ERO mouse facility or from Jackson Labs for all studies. All experiments were performed in accordance with the guidelines approved by the University of Texas MDACC Institutional Animal Care and Use Committee. Three days after the intravenous introduction of 1 × 10^6^ MOLM13 or 0.5 × 10^6^ MV411 (FLT3‐ITD‐positive acute myeloid leukemia) cells transduced with luciferase, leukemia engraftment was confirmed via bioluminescent imaging. In all experiments, doxorubicin (2 mg/kg or 1 mg/kg in a TRF experiment) or a PBS control was delivered twice weekly via intravenous injection. Concomitant with treatment, mice were fed a low‐sucrose diet (7% sucrose), low‐fat diet (10%), (D12450H) a low‐fat (30%)/low‐sucrose (10%) diet (D12492), high‐fat diet (60%) (D12451), high‐sucrose diet (30%), or normal chow (ordered from Research Diets) for the duration of the experiment. Low‐ and high‐sucrose feeding in mice was achieved through drinking water replacement for the duration of the experiment. Leukemic burden was tracked weekly via bioluminescent imaging using an IVIS Lumina XR Imaging System after injection with 200 μL D‐luciferin subcutaneously. TRF was implemented using the PhenoMaster system (TSE Systems, Bad Homburg, Germany). The automatic feeder was open from 7 PM to 7 AM for cages set for TRF paradigm. Cages in the control (24 food access) had the feeder open 24 h a day. Feeding behavior, drinking behavior, locomotion, and energy expenditure was measured for 12 days using the PhenoMaster system (TSE Systems, Bad Homburg, Germany). The following parameters were measured: daily food consumption [g], daily water consumption [mL], S. flow [L/min], Ref. O_2_ [%], Ref. CO_2_ [%], O_2_ [%], CO_2_ [%], VO_2_ [mL/h/kg], VCO_2_ [mL/h/kg], RER, wheel rotations: (i) right [Rot], (ii) left [Rot], (iii) sum R + L [rot], sumtime [min], sumruns, maxspeed [rpm], avgspeed [rpm], and maxlength [s]. Measurements were recorded every 45 min over 12 days continuously.

### 2.2 Observational study of diet receptivity

After obtaining informed consent and assent, 12 (nine of which were diagnosed with standard risk B‐ALL) study participants were recruited in Houston, Texas from the MD Anderson Cancer Center Children's Cancer Hospital in accordance with approval from the Institutional Review Board of the University of Texas MD Anderson Cancer Center (2010‐0654). Eligibility criteria included children being school aged (5–17), pediatric cancer patients who were currently undergoing treatment, and ability to read and speak English. Each participant attended in‐person sessions wherein they tasted five low‐fat prepared foods and five low‐sucrose prepared foods prepared by the Bionutrition Research Core at MD Anderson Cancer Center using recipes from the At The Table online cookbook.^8^ Fat and sucrose content of prepared foods was analyzed using the University of Minnesota Nutrition Data System for Research (NDSR) Software, Version 2011. During the sessions, a single patient, and their caregiver (if available) was led through the food tasting by research staff using a semi‐structured interview guide to conduct a Qualitative Intensive Interview. Sessions were audio‐recorded and professionally transcribed using NVIVO, a qualitative software analysis program. Initial ratings were classified into three categories, “Liked,” “Neutral,” or “Disliked.” Transcriptions were reviewed using a rapid qualitative analysis approach^9^ guided by our research objective to ascertain actionable recommendations for improvement in anticipation of a larger pilot study.

### 2.3 Cell lines, chemicals, and reagents

Human AML cell lines, MOLM13 (ACC‐554), Kasumi‐1 (CRL‐2724), and MV411 (CRL‐9591) were purchased through ATCC. All cells were grown in RPMI‐1640 media containing 10% fetal bovine serum (Genesee Scientific, California, USA) with supplemental 1% pen/strep (HyClone, Utah, USA) and 1% L‐glutamine (Sigma‐Aldrich, Missouri, USA) and monitored for mycoplasma with MycoAlert PLUS (Lonza, Basal, Switzerland) and authenticated to confirm identity. Doxorubicin and daunorubicin (MD Anderson Cancer Center Pharmacy), nobiletin (Sigma‐Aldrich, Missouri, USA), D‐luciferin (GoldBio, Missouri, USA), DMSO (Sigma‐Aldrich, Missouri, USA), and resazurin sodium salt (Sigma‐Aldrich, Missouri, USA) were purchased. Resazurin fluorescence (excitation = 530–570 nm and emission = 590–620 nm) was measured using a Clariostar microplate reader.

### 2.4 Western blot

Spleen or liver tissues were homogenized in RIPA lysis buffer (50 mm Tris–HCl (pH 8.0),150 mM NaCl, EDTA 5 mM, MgCl_2_ 15 mM protease inhibitors, and NP‐40 1%, 1 mM PMSF, 1 mM NaF, 400 mM NAM, and 3.3 mM TSA) for 15 s, using a MagNA Lyser (Roche, IN, USA). Samples were spun at 10,000 *g* for 10 min to eliminate insoluble material. The total protein levels of the lysates were determined using the bicinchoninic acid method. Protein extracts were analyzed using 8% sodium dodecyl sulfate–polyacrylamide gel electrophoresis, then transferred to the nitrocellulose membrane before staining with primary antibodies. Secondary antibodies conjugated to horseradish peroxidase and enhanced chemiluminescence substrate were used to detect.

### 2.5 Oxidative stress measurements

Levels of intracellular peroxides and superoxides were measured in mouse peripheral blood mononuclear cells after blood was collected via cardiac puncture at the conclusion of in vivo experiments. Red blood cells were lysed via ACK lysis buffer twice and washed twice. Hydroethidium (HE) (Thermo Fisher, Massachusetts, USA) and dichlorofluoroscein (DCF) (Thermo Fisher, Massachusetts, USA) stains were used at 10 μM and 10 μM, respectively, to stain the cells for 30 min at 37°C in the dark. The cells were then washed with PBS and resuspended in 300 μL PBS for acquisition on a BD Biosciences Fortessa flow cytometer.

### 2.6 CyToF analysis

Spleens were harvested from mice and the tissue was homogenized. Cells were washed twice with PBS and counted with the ViCell XR analyzer and resuspended in wash buffer (0.5% BSA in PBS). A cell surface antibody mix was added to each sample and put on the shaker for 1 h at room temperature. Cells were then washed three times to remove residual antibodies and 1 μM Pt198 (Fluidigm), a live/dead stain, was added to each sample for 5 min on the shaker at room temperature. Cells were washed three times, then 4% paraformaldehyde was added to each sample and were incubated for 15 min at room temperature. Cells were washed once, then 1 mL 100% methanol was added to each sample overnight. After one wash, intracellular antibody mix was added to each sample with incubation for 1 h on the shaker at room temperature. Cells were washed two times, then Ir‐intercalator‐193, 125 μM (Fluidigm, California, USA) was added to each sample with a final dilution of 1:2000. Samples were analyzed with the Fluidigm Helios CYTOF Mass Cytometer.

### 2.7 Transfection

Cells were centrifuged at 300 *g* for 5 min to remove the culture medium and resuspended in PBS for 5 min. Around 1 × 10^6^ cells in 100 μL electroporation buffer (Lonza #VCA1003) were prepared and mixed with 2 μg of linearized (PeGFP/PeGFP‐bmal1) DNA. The mixture was added into a certified cuvette and placed into the BIO RAD Gene Pluser Xcell electroporation stage with 1000 μF under 155 V. The mixture was then transferred to a six‐well cell culture plate and incubated at 37°C in a humidified atmosphere containing 5% CO_2_.

### 2.8 Statistical analysis

All statistical tests are *t*‐tests with multiple comparisons or ANOVA tests. GraphPad Prism version 7 was used for all other statistical analyses unless otherwise indicated. Two‐tailed *t*‐test for experiments with two groups; for experiments with two groups and multiple time points, multiple *t*‐tests were performed with multiple comparisons using the Holm–Sidak method with an alpha of 0.05, and then adjusted *p*‐value was used to determine statistical significance. ANOVA was used for mouse experiments with three or more groups. For experiments with multiple time points, ANOVA with repeated measures was performed. In the observational study, transcriptions were reviewed using a rapid qualitative analysis approach.^9^ Demographic information was summarized using descriptive statistics. Associations between categorical variables were examined by chi‐squared or Fisher's exact tests and computations were carried out in SAS 9.3 (SAS Institute Inc., Cary, NC, USA).

## 3 RESULTS

**FIGURE 1:**
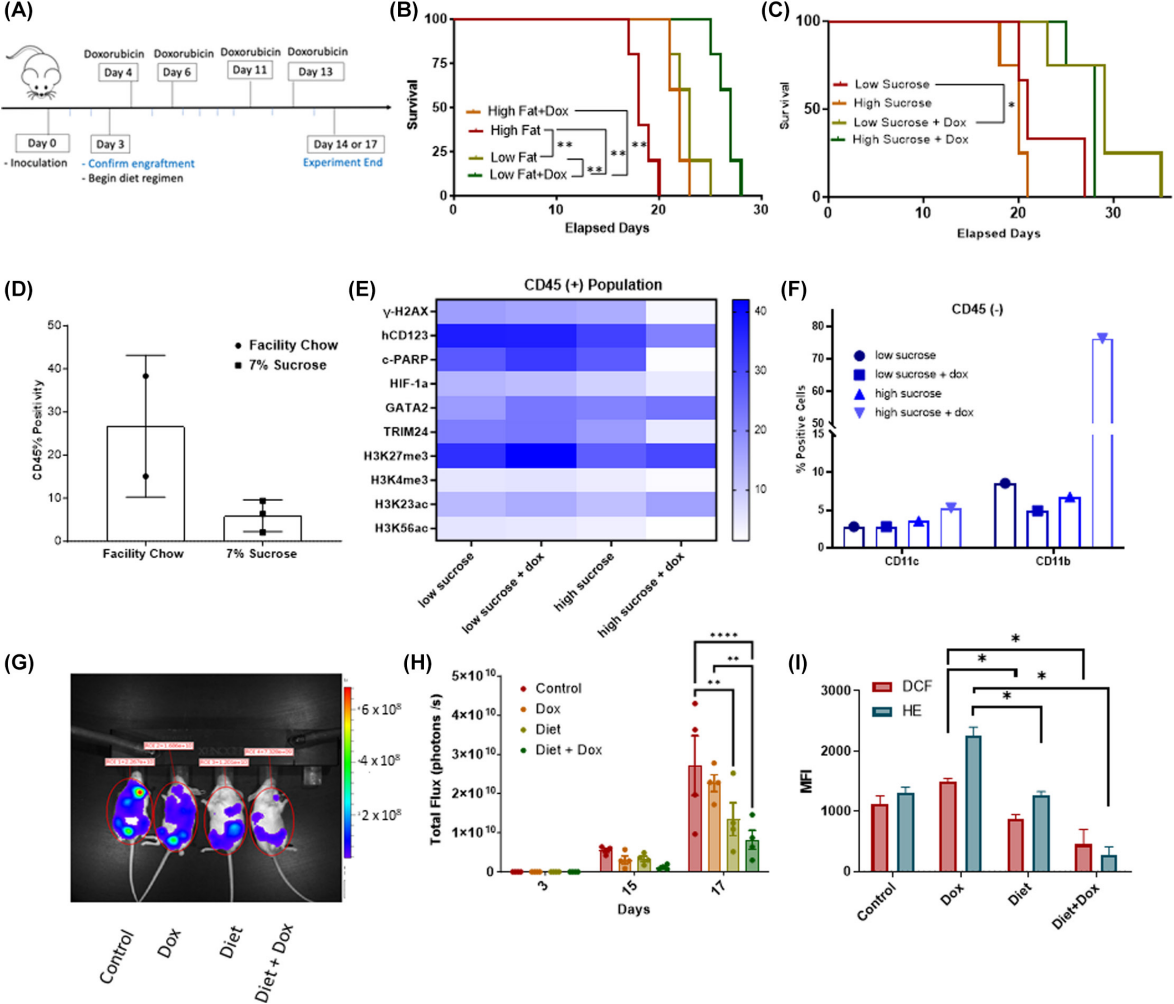
Diet interventions combined with treatment in orthotopic xenograft FLT3‐ITD‐bearing AML mouse models. (A) Schematic of experimental set up for all animal experiments. SCID mice were inoculated with 1 × 10^6^ MOLM13 cells on Day 0. On Day 3, bioluminescent imaging was conducted to measured tumor burden. The mice were normalized for equal average bioluminescence into four groups: diet regimen, diet regimen plus doxorubicin (dox) treatment, normal chow, and normal chow plus dox treatment. Diet regimens began on Day 3 and a representative doxorubicin dosing schedule is shown in the figure. (B) Kaplan–Meier survival curve for low‐fat diet intervention (*n* = 5 per group) (C.) Kaplan–Meier survival curve for low‐sucrose intervention (*n* = 3 for low‐sucrose group, *n* = 4 all other groups). (D) Percentage of CD45‐positive cells in mouse spleens 21 days after leukemia engraftment in mice fed facility chow or a low‐sucrose diet. (E) Heat map of protein expression in CD45‐positive spleen cells as measured by CyToF in MOLM13‐bearing mice fed a low‐ or high‐sucrose diet in the absence or presence of doxorubicin treatment. (F) Quantification of the percentage of cells that positively stain for mCD11c and mCD11b in CD45‐negative cell populations. (G) Representative image of IVIS bioluminescent imaging of mice with diet intervention, diet + dox, normal chow, and normal chow + dox with representative ROI analysis. (H) Leukemic burden was measured via total flux on Day 3, Day 15, and Day 17 of the experiment. (I) Quantification of the fluorescence intensity of DCF and HE staining of peripheral blood mononuclear cells from each control or treatment group on Day 17 is shown. DCF and HE stains are used for measures of superoxides and peroxides, respectively. The bar graphs in H and I depict the mean value ± standard error of the mean. A two‐way ANOVA test was used in Figures H and I. *p < 0.05, **p < 0.01, ***p < 0.001.

The impact of diet on AML patients bearing a FLT3‐ITD mutation is unclear. However, several studies have documented poor diet quality, consistent with western dietary patterns with high consumption of fat, sugary drinks, and refined carbohydrates in AML patients. Furthermore, high‐fat mouse chow has been shown to accelerate AML growth in vivo, but these studies have not focused on the FLT3‐ITD mutation. We sought to understand how modulating sucrose and fat content in mouse chow would impact therapy response in FLT3‐ITD AML‐bearing orthotopic mouse models. Because diet modification in the context of therapy has more clinical relevance than modulation of diet in the absence of treatment, we studied the impact of diet composition modulation on anthracycline treatment response. Once leukemia engraftment was confirmed, mice were separated into groups for diet modification in the presence or absence of doxorubicin treatment (Figure 1A). Figure 1B shows overall survival of mice fed a high‐fat diet (60% calories from fat), or a low‐fat diet (10% calories from fat), in the presence or absence of doxorubicin treatment. Interestingly, leukemia‐bearing mice that were fed a low‐fat diet had significantly longer survival (*p* < 0.05) than mice fed a high‐fat diet (Figure 1B). Mice in the low‐fat + doxorubicin treatment group showed extended survival relative to all the groups (*p* = 0.0018) (Figure 1B). A similar experimental design was employed to assess the impact of low‐sucrose (7% sucrose), or high‐sucrose diet (30%), in AML FLT3‐ITD‐bearing mice and found that survival appeared extended in the low‐sucrose + doxorubicin treatment group, but did not reach statistical significance (*p* = 0.2) (Figure 1C). CD45 positivity in spleen cells from these mice indicated that mice fed low‐sucrose chow showed lower leukemic burden (Figure 1D).

We also sought to gain insight into proteomic changes that arose from diet modulation. For mice on the low‐sucrose versus high‐sucrose diet regimen, peripheral blood mononuclear cell samples were analyzed via mass cytometry. Human CD45‐positive cells from mice fed low‐sucrose chow and treated with doxorubicin, showed increased cleaved PARP expression compared to cells from mice fed high‐sucrose chow and treated with doxorubicin (Figure 1E). In CD45‐negative cells, CD11b positivity was lower in low‐sucrose treatment regimens (Figure 1F), whereas the low‐sucrose and doxorubicin‐treated mice had a higher population of CD11c‐positive cell populations. This suggests that changes to sucrose concentration in diet correlates with changes in macrophage/dendritic cells populations which in turn are associated with decreased leukemia burden observed in the anthracycline treated low‐sucrose versus normal chow fed mice.

As the low‐sucrose and low‐fat diet intervention's impact on overall survival trended toward significance, we tested whether combining a low fat (10%)/low sucrose (10%) intervention would augment doxorubicin response in FLT3‐ITD AML‐bearing mice. Mice on the low‐fat/low‐sucrose regimen, with or without doxorubicin, had significantly lower tumor burden compared to leukemia‐bearing mice that were fed normal chow control on Day 17 (Figure 1G,H). The diet plus doxorubicin treatment mice had significantly lower tumor burden compared to doxorubicin alone, indicating the diet intervention augmented doxorubicin efficacy. Doxorubicin treatment is known to increase oxidative stress in PMBCs^10^, ^11^ which is linked to late effects of treatment. In order to determine if diet modulation influenced redox status in vivo, we measured superoxide levels and intracellular peroxide levels production in peripheral blood mononuclear cells isolated from blood samples taken from mice on Day 17. Figure 1I shows that fluorescence of both DCF (dichlorofluoroscein), which measures intracellular peroxide levels, and HE (hydroethidium) which measures intracellular superoxide levels, were significantly lower in the in the diet‐plus‐doxorubicin group compared to the doxorubicin‐plus‐normal‐chow group. This indicates that the diet intervention reduces oxidative stress while simultaneously augmenting efficacy of doxorubicin.

**FIGURE 2:**
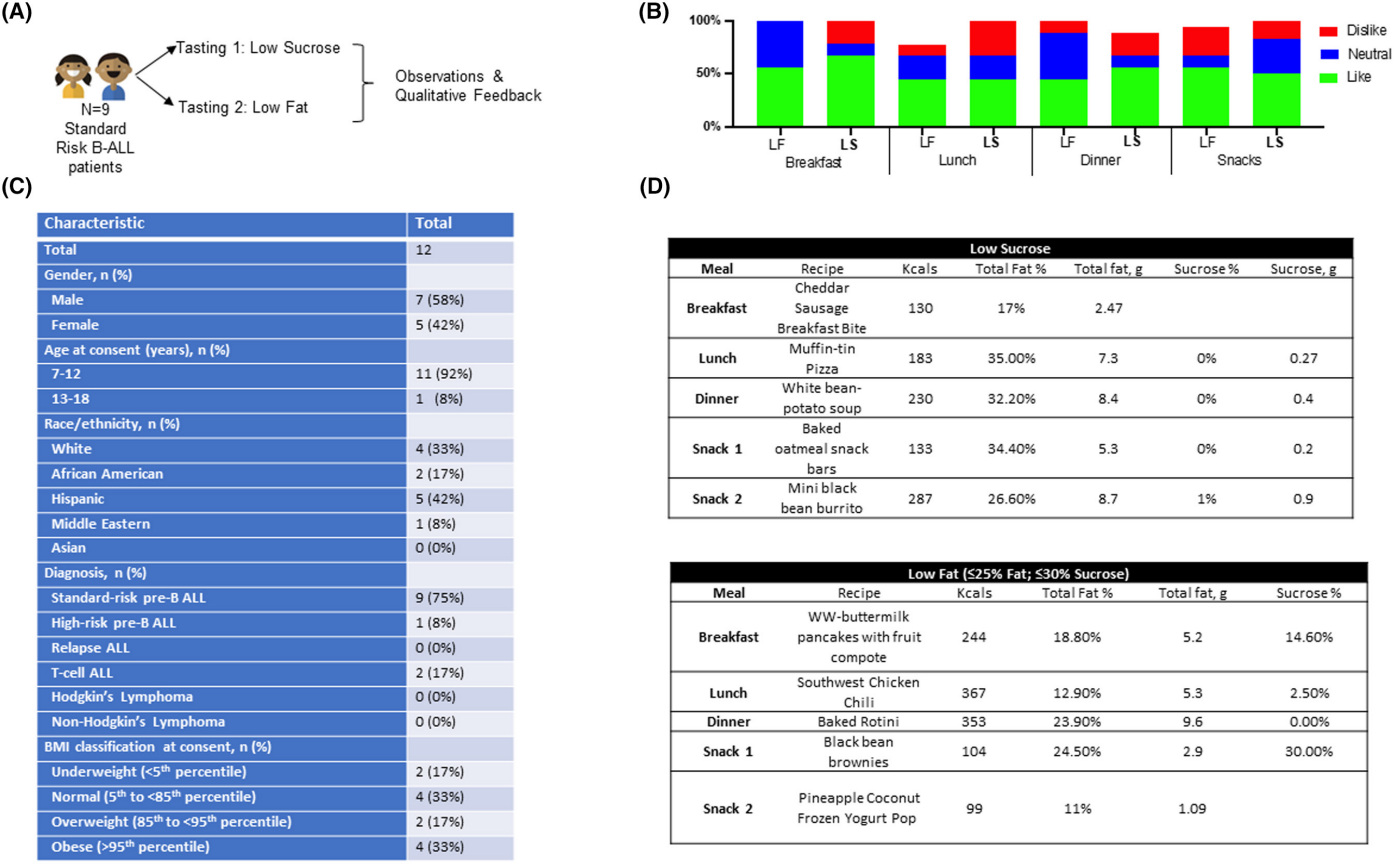
Leukemia patient receptivity to low‐fat and low‐sucrose foods. (A) Schematic of tasting study for pediatric participants on active treatment for leukemia or lymphoma (*n* = 9). (B) Quantification of percentages of pediatric participants who liked, disliked, or felt neutral about the low‐fat or low‐sucrose meals presented to them. (C and D) Tables showing the macronutrient content of the low‐sucrose (C) and low‐fat (D) meals tasted by the study participants.

Our data point to a novel finding: that diet composition augments doxorubicin efficacy in FLT3‐ITD AML in vivo, indicating that a diet based behavioral modification concurrent with leukemia treatment may have clinical application. However, an important question remains concerning the feasibility of implementing a diet change for pediatric patients actively undergoing treatment. Therefore, we conducted an observational tasting study with nine pediatric patients diagnosed with leukemia and undergoing treatment to study the receptivity of patients to a low‐fat or low‐sucrose intervention matching the macronutrient distribution in the mouse studies (Figure 2A). The majority >90% of these patients were within the 7‐ to 12‐year old age range (Figure 2C). Over the course of two separate tasting session, participants were presented with low‐fat (LF) or low‐sugar (LS) meals and were asked to rate their liking, neutrality or dislike of the meals (Figure 2B). Overall, the participants reported positive feedback for low‐sucrose and low‐fat meal options and patients preliminarily indicated willingness to maintain a diet intervention during treatment composed of recipes that were low sucrose or low fat (Figure 2D). All recipes are freely available on a website created by our group,^12^ and both participants and their parents were provided print materials directing them to the e‐cookbook site.

**FIGURE 3:**
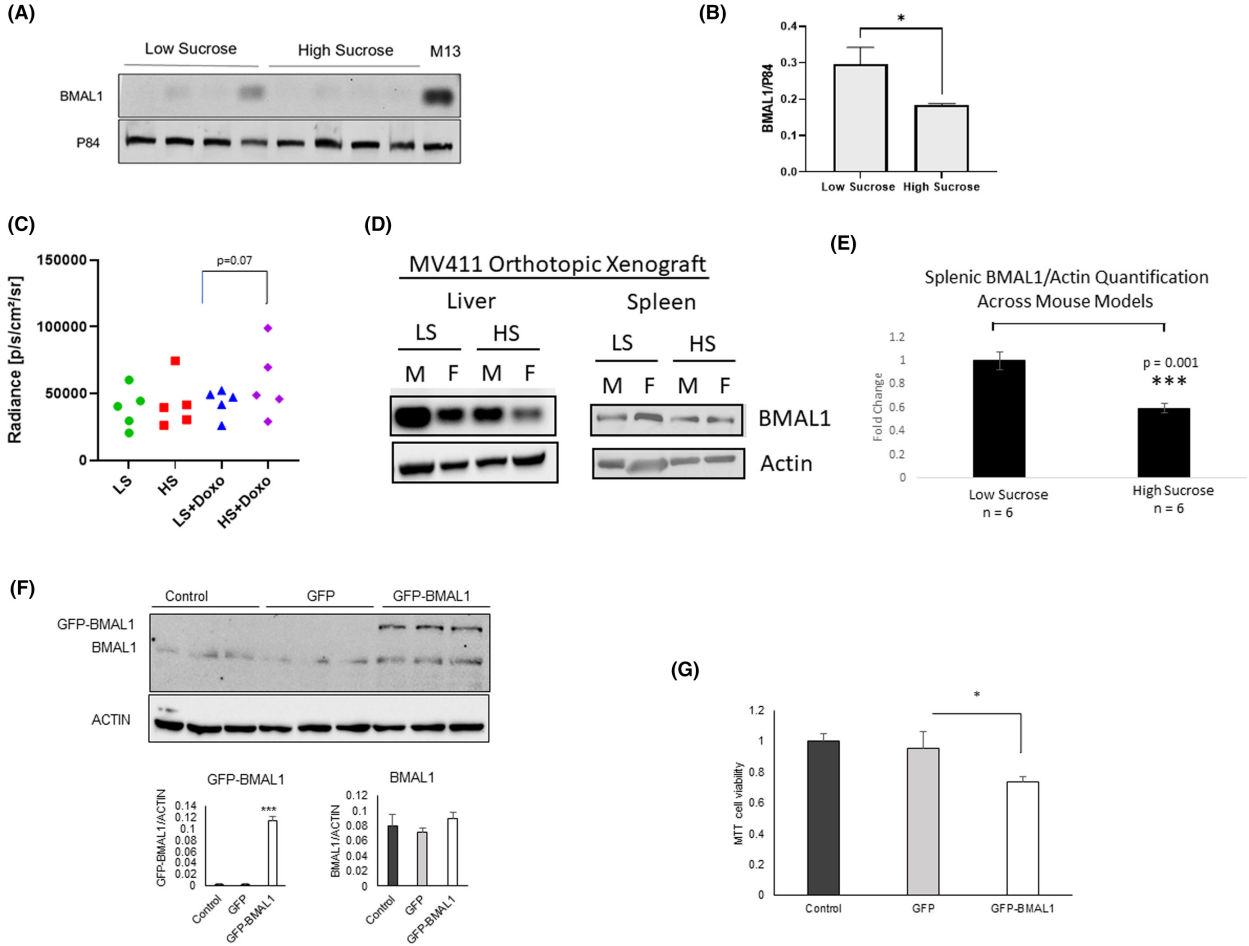
Increased BMAL1 in low‐sucrose diet fed mice and effects of BMAL1 overexpression in AML cell lines. (A) Western blot images show spleen samples isolated from low‐sucrose fed mice and high‐sucrose fed mice on Day 17 of the experiment probed for BMAL1 and P84 as a loading control. (B) Quantification of the western blot image is shown, with BMAL1 signal normalized to P84 signal. (C) Quantification of tumor burden in MV411‐luciferase‐bearing mice exposed to low‐sucrose water (LSW), high‐sucrose water (HSW), with or without 2 mg/kg doxorubicin treatment (Doxo). Data shown are after 7 days of diet change and doxorubicin treatment. (D) Western blot images of BMAL1 and Actin expression in liver and spleen from male (M) and female (F) low‐sucrose (LS) and high‐sucrose (HS) fed mice bearing the MV411 model. (E) Quantification of BMAL1 from western blots from spleens of MOLM13 and MV411 mice exposed to low‐ or high‐sucrose diet. (F) Western blot images showing expression of BMAL1 and GFP‐BMAL1 in MOLM13 cells transfected with GFP loading vector or BMAL1. Quantification of GFP‐BMAL1 and BMAL1 is depicted graphically below western blot. (G) Cytotoxicity of BMAL1 overexpression from experiment described in (F). Forty eight hours post transfection, MTT solution was added to cells and absorbance was measured as an indirect reading of cell viability.

Diet is a regulator of circadian rhythmicity,^13^, ^14^ and BMAL1 has been linked to AML stem cells, but has not been interrogated in the context of diet and AML treatment. Given that circadian protein function in the context of leukemia malignancy is not well defined, we also sought to analyze changes in circadian protein expression in spleen samples in the low‐sucrose versus normal chow experiments. Mice on a low‐sucrose diet had significantly higher expression of BMAL1 compared to the high‐sucrose group (Figure 3A,B). In order to extend these data to a second AML mouse model, we utilized MV411‐luciferase labeled cells orthotopically engrafted into male and female NSG mice via tail vein injection. In vivo imaging was conducted on day 3 to insure leukemia cell engraftment, at which point, mice were divided into groups of mice drinking low‐sucrose water (LSW; 7% sucrose) or high‐sucrose water (HSW; 30%) with attention to mean leukemia burden as being equivalent across all groups. Treatment with doxorubicin was given twice weekly at a dose of 2 mg/kg. Data shown in Figure 3C indicate that leukemia burden was lower in mice exposed to low‐sucrose drinking water and doxorubicin therapy (LSW + Doxo) than mice exposed to high‐sucrose drinking water and doxorubicin (HSW + Doxo), although this did not reach statistical significance (*p* = 0.07). We examined BMAL1 expression by western blotting in male (M) and female (F) mice from these groups (Figure 3D) and found higher BMAL1 expression in liver and spleen of low‐sucrose fed animals. This effect was more pronounced in female mice and was consistent with data shown in the MOLM13 mouse model (Figure 3A). When combined together, BMAL1 expression data in spleens analyzed from western blotting by densitometry across MOLM13 and MV411 mouse models showed that low‐sucrose diet increased BMAL1 expression significantly (*p* = 0.001) compared to high‐sucrose diet (Figure 3E). To determine the effects of increased BMAL1 expression in AML cells, MOLM13 cells were transfected with GFP‐BMAL1 and showed significantly reduced cell viability compared to control construct transfected cells (Figure 3F,G). This suggests that high BMAL expression contributes to cytotoxicity in AML cells.

**FIGURE 4:**
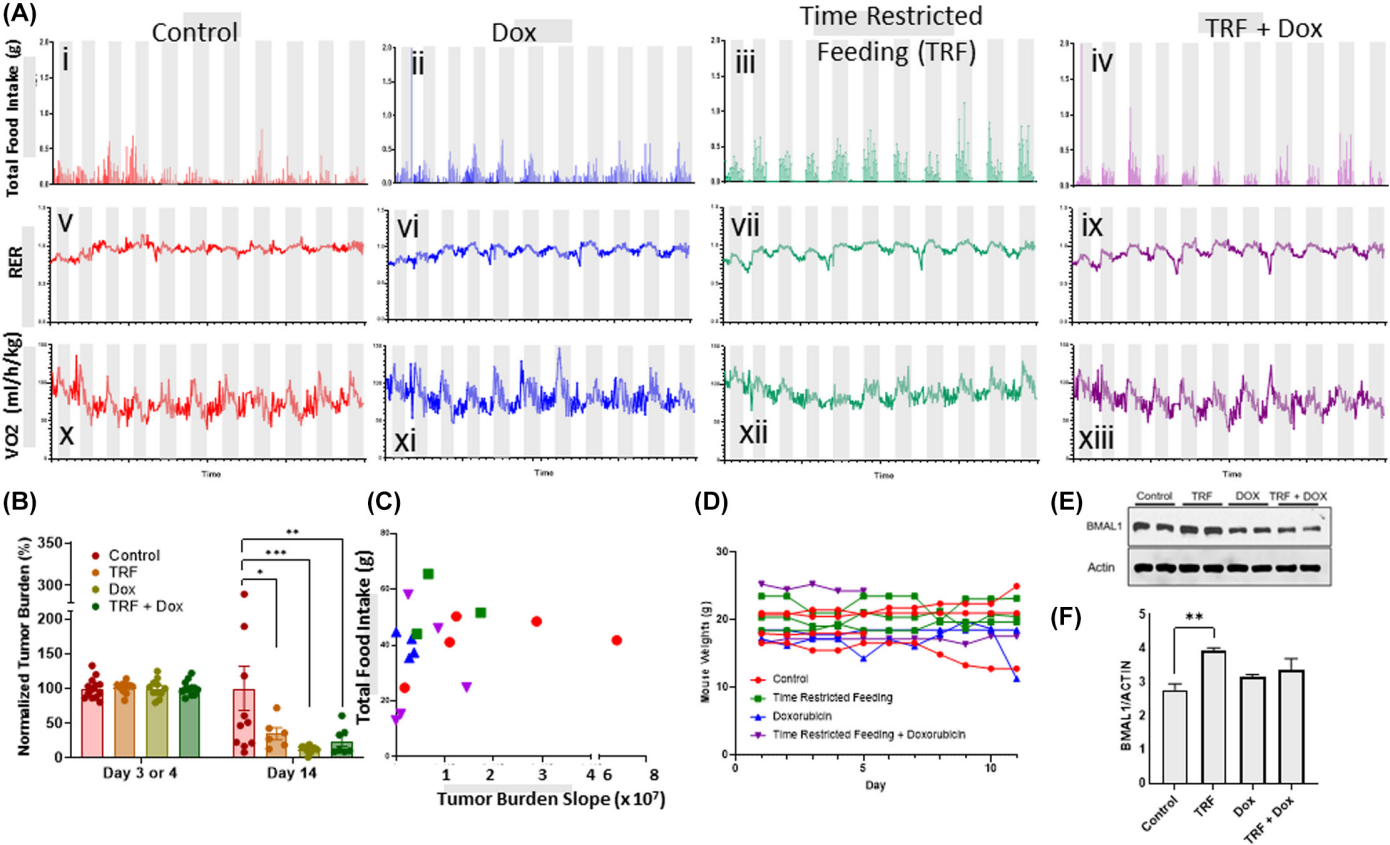
Effects of TRF of AML growth in vivo. (A) Mice were inoculated with 1 × 10^6^ MOLM13 cells on Day 0 of the experiment. On Day 3, engraftment was confirmed via bioluminescent imaging, and then mice were normalized into groups based on total flux (photons/s) measured via IVIS imaging. Mice were then moved into single housed metabolic cages which measured total food intake in grams, oxygen consumption (VO_2_) and carbon dioxide produced (CO_2_) at 45‐min intervals. Graphs i–iv depict mean values of total grams of food intake per group for each 45‐min interval over 11 days (*n* = 3–5 per group). Graphs v–ix depict mean values of RER per group for each 45‐min interval over 11 days (*n* = 3–5 per group). Graphs x–xiii depict mean values of oxygen consumption per group for each 45‐min interval over 11 days (*n* = 3–5 per group). (B) Leukemic burden as measured by IVIS imaging on Day 3 and Day 14 of the experiment is depicted. Each data point is normalized to the mean total flux value for that group. Total food intake and total water intake over the entire 14 days spent in cages was recorded for each individual mouse. (C) The tumor growth slope value and food intake for individual mice is shown above, with each data point representing an individual mouse. (D) Mouse weight over the duration of the experiment. (E) Western blot images show spleen samples isolated from mice in each treatment and control group on Day 14 of the experiment probed for BMAL1 and actin as a loading control. (F) Quantification of the Western blot image is shown, with BMAL1 signal normalized to actin signal. The bar graphs in M and Q depict the mean value ± standard error of the mean. A one‐way or two‐way ANOVA test (dependent on number of groups) was used to determine significance in M and Q. *p < 0.05, **p < 0.01, ***p < 0.001.

Timing of diet, and various paradigms of TRF,^15^, ^16^ are known to influence circadian rhythmicity, but have not been examined in leukemia models. We assessed the impact of TRF on leukemia burden and treatment response, as measured in vivo. Mice were singly housed in metabolic cages and after leukemia engraftment was confirmed, given 24‐h access to food (ad libitum) or underwent TRF: 12‐h access to food during their active phase. As with experiments shown in Figure 1, doxorubicin was administered to mice after engraftment was confirmed and continued twice weekly. After 11 days on this diet paradigm, the mice in the TRF group did not have significant differences in total caloric intake (Figure 4A(i‐iv)). Further, 24‐hr. RER (Figure 4A(v‐ix)) and oxygen consumption (Figure 4A(x‐xiii)) patterns suggest that circadian rhythmicity was sustained in all groups. However, noninvasive in vivo imaging of leukemia progression showed significantly lower tumor burden in TRF mice compared to the control mice (Figure 4B). Doxorubicin efficacy remained robust and was not compromised by the TRF regimen. The effect of TRF on leukemia burden did not rely on total food intake or weight status of mice, as neither of these variables were significantly different across groups (Figure 4C,D). In assessing protein expression from total spleen tissue, we found that spleen from TRF‐treated mice showed significantly higher BMAL1 expression compared to spleen tissue from control mice (Figure 4E,F).

**FIGURE 5:**
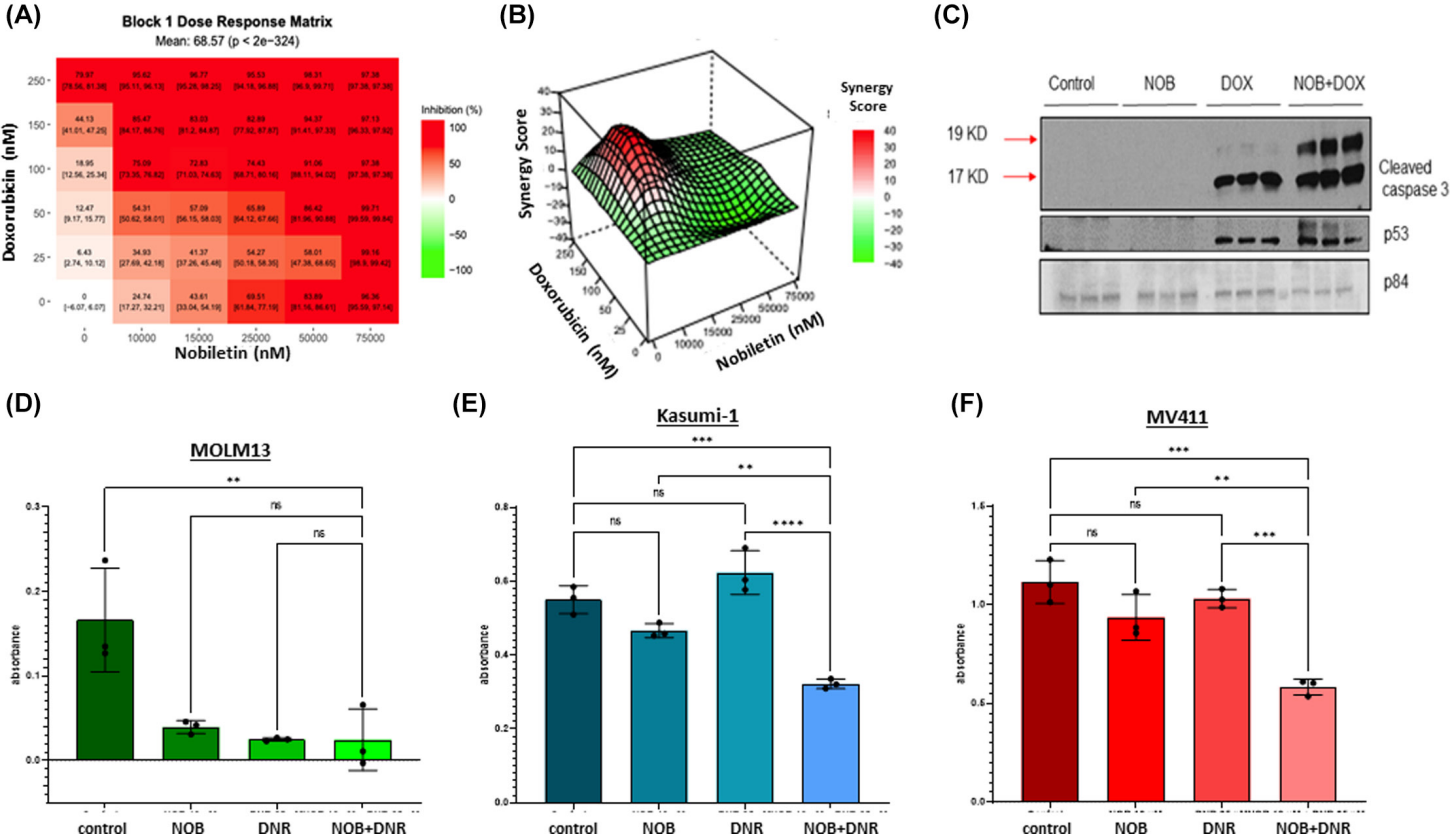
Nobiletin enhances anthracycline‐induced AML cytotoxicity. (A) MOLM13 cells were treated with varying doses of nobiletin and/or doxorubicin for 48 h. Viability was measured via Alamar Blue assay and percent viability, or percent inhibition, is depicted on the graph above made by a web‐based application, Synergy Finder.^19^, ^20^ (B) Synergy was calculated by the software and the ZIP synergy score at different combination concentrations is shown in the 3D plot (data shown are the mean from one experiment and one additional replicate done with similar result not shown). (C) Western blot stained for cleaved caspase 3, p53, and normalizing protein p84 shown in protein lysates from MOLM13 cells treated with nobiletin, doxorubicin, or a combination for 72 h. (D) MOLM13 cells, (E) Kasumi‐1 cells, and (F) MV411 cells were seeded in a 96‐well plate and treated with 10 μM nobiletin, 25 nM daunorubicin, or a combination of the two for 72 h. MTT solution was added to each well and absorbance was measured as an indirect measure of cell viability. One‐way ANOVA was conducted to determine significance. The bar graphs in C, D, and E depict the mean value ± standard error of the mean. *p < 0.05, **p < 0.01, ***p < 0.001.

Finally, we sought to determine whether pharmacological modulation of BMAL1 could influence AML cell viability when combined with doxorubicin. Previous reports indicate that the natural flavonoid, nobiletin, indirectly upregulates BMAL1 protein expression, by acting as an agonist of retinoic acid receptor‐related orphan receptor alpha (RORα) agonist.^17^, ^18^ MOLM13 cells treated with nobiletin in a dose dependent manner, showed that the small molecule decreased cell viability with an IC50 of 23 μM (Figure 5A). When combined with doxorubicin treatment in vitro, viability was significantly lower compared to either treatment alone, indicating that combining BMAL1 modulation with doxorubicin may provide a synergistic effect (Figure 5A,B). Synergy analysis using the Zero interaction potency (ZIP) model^19^, ^20^ (Figure 5B) confirmed the combination's concerted effects, and western blotting of MOLM13 cells treated with the combination of nobiletin and doxorubicin showed higher levels of cleaved caspase‐3 than either agent alone, indicating that cell death was enhanced by the combination (Figure 5C). This was further extended to additional AML cell lines, Kasumi‐1, and MV411, treated with the combination of daunorubicin (another anthracycline) and nobiletin (Figure 5D–F).

## 4 DISCUSSION

Although much progress has been made in improving treatment outcomes for patients with an acute myeloid leukemia (AML) diagnosis, certain subsets of AML, such as those with a FLT3‐ITD mutation, have poor treatment advances and low survival rates. There is a significant need to improve treatment outcomes, without increasing toxicity of treatment. Given that malignant cells can be distinguished from their healthy cell counterpart based on changes in metabolism (e.g., the increased dependence on anaerobic glycolysis, known as the Warburg effect), many previous studies have sought to understand how extrinsic factors, like diet, may impact cancer progression. For example, many studies have found that diet high in fat or sugar can lead to more aggressive cancer progression in breast cancer,^21^, ^22^ colon cancer,^23^, ^24^ and melanoma.^25^ However, little work has addressed this issue in molecular subsets of AML, and none have focused on FLT3 mutant disease.

A study conducted in immunocompetent MLL‐AF9 knock‐in mice fed a high‐fat diet (42% calories from fat) for 4 weeks showed accelerated leukemia growth compared to mice on a low‐fat diet, and this was correlated with FLT3 receptor phosphorylation.^2^ Many studies such as this have identified high‐fat diet to worsen leukemia progression,^3^ but none have implemented a low‐fat diet as a possible intervention to improve treatment. One study identified AML cells to be high in amino acids such as methionine, cysteine, and lysine, and showed that depleted methionine from the diet in vivo slowed leukemic progression.^26^ However amino acid depletion diets are difficult to implement in clinical settings, as this requires a very specific diet formulation and strict adherence.

Strengths of our study include the finding that diets consistent with healthy lifestyles can influence AML FLT3‐ITD growth in vivo and anthracycline based chemotherapy efficacy. In our mouse models, the diets were adopted after leukemia engraftment, suggesting that diet change during treatment may be beneficial. Importantly, the diet interventions we employed in the mouse models are feasible for clinical testing. Our pilot tasting study showed that macronutrient matched whole food recipes were well received by pediatric patients undergoing treatment for leukemia. This indicates that diet interventions centered around macronutrient modification combined with treatment are feasible. This study is meant to be a preliminary study, and as such only included nine pediatric acute leukemia patients with standard risk disease, although a total of 12 patients were studied, to show feasibility of implementing a diet intervention for pediatric patients on a larger scale. A future study should be done with a larger cohort to measure for improved outcomes in 5‐year survival and toxicity.

Our findings show that efficacy of anthracyclines, which are used globally for AML therapy, is enhanced by low‐fat/low‐sugar diets. The FLT3‐ITD expressing subset of AML was the focus of our investigations due to poor outcomes seen in patients. Although targeted therapies for FLT3 mutant AML are available with several agents approved by the FDA, the access to these agents globally and even within nonurban domestic regions is uncertain, therefore these behavioral modifications may have broad impact in underserved populations. Further studies should explore how macronutrient diet modifications impact FLT3 target therapy efficacy or combined treatment regime efficacy.

In our investigation, we also sought to explore how extrinsic factors beyond diet composition may impact leukemia progression. Many studies have implicated circadian rhythmicity dysregulation as a possible contributor to cancer progression.^27^, ^28^, ^29^ Restricted feeding, a well‐known regulator of circadian rhythm, and caloric restriction^30^, ^31^ have been shown to be beneficial in certain cancer models and metabolic disease,^16^ but has not yet been explored in leukemia models. Interestingly, core clock circadian proteins have been implicated as being both positive and negative regulators of tumorigenesis.^29^, ^32^, ^33^, ^34^ For example, knocking out various clock genes has been showed to promote lung tumorigenesis,^28^ while knocking out various clock genes led to disease regression in a glioblastoma model.^35^ One study found that both CLOCK and BMAL1 proteins are required for leukemia stem cell growth,^7^ and disruption of the circadian clock leads to LSC differentiation, thereby inhibiting progression.

Therefore, our study sought to understand how a circadian clock intervention would impact AML progression. We report, for the first time, that TRF does not limit anthracycline efficacy and appears to exert autonomous slowing of leukemia progression in vivo. Our results also suggest that BMAL1 modulation has therapeutic implications in AML, as overexpression causes cell death, and pharmacological modulation shows synergy with anthracyclines. The previous study from Ebert et al. focused on leukemia stem cells' genetic composition,^7^ while our study focuses on FLT3‐ITD mutant AML cell lines derived from human samples. This underscores the complexity of BMAL1's role in AML progression. Our study is meant to preliminarily identify BMAL1 as a potential therapeutic target. Deeper analysis of BMAL1 activity, its binding partners and cell type specific expression, in leukemia is warranted. Further explanation of other clock proteins and circadian oscillation changes role in leukemia progression should also be explored.

Strengths of our study include the first report of low‐sucrose diet and timing affecting leukemia progression in a particularly refractory subtype of AML, and being accompanied by an increase in BMAL1 expression. Furthermore, this is the first report, to our knowledge, of BMAL1 overexpression inducing cell death in AML, which can be mimicked with a pharmacological inhibitor. Weaknesses of our study include the use of an orthotopic xenograft rather than a PDX or syngeneic model for FLT3 mutant AML, the small size of our patient cohort for diet testing, the focus on BMAL1, and the off‐target effects of nobiletin and its varied effects across AML cell lines as a single agent. However, all of these limitations are launching points for future studies. Collectively, our results suggest that interventions modulating diet quality and diet timing together with leukemia therapy are effective and feasible treatment strategy as indicated by our parallel animal‐human studies. Further work should explore whether these interventions augment other chemotherapies and targeted therapies, and the associated molecular mechanisms by which these interventions regulate leukemia growth and progression. In addition, our study focused on FLT3‐mutant AML models. These studies should be expanded to other AML mutant types and these experiments should be repeated in PDX models, to understand whether these observations are true broadly for leukemias, or if this is specific to the FLT3‐ITD aberrant signaling pathway.

## AUTHOR CONTRIBUTIONS

**Megan Rodriguez:** Formal analysis (lead); investigation (supporting); writing – original draft (lead). **Baharan Fekry:** Data curation (equal); formal analysis (equal); validation (equal). **Brianna Murphy:** Investigation (supporting). **Mary Figueroa:** Investigation (supporting). **Tiewei Cheng:** Investigation (supporting); validation (supporting). **Margaret Raber:** Formal analysis (supporting); investigation (supporting); methodology (supporting). **Lisa Wartenberg:** Formal analysis (supporting); investigation (supporting); project administration (supporting). **Donna Bell:** Investigation (supporting). **Lisa Triche:** Investigation (equal). **Karla Crawford:** Data curation (supporting); investigation (supporting); project administration (supporting). **Kendra Allton:** Formal analysis (supporting); investigation (supporting); methodology (supporting). **Jaime Tran:** Investigation (supporting). **Christine Ranieri:** Investigation (supporting); resources (supporting). **Marina Konopleva:** Resources (supporting). **Michelle Barton:** Resources (supporting). **Cesar Nunez:** Investigation (supporting); project administration (supporting). **Kristin Eckel‐Mahan:** Conceptualization (supporting); funding acquisition (supporting); investigation (supporting); methodology (supporting); project administration (supporting). **Joya Chandra:** Conceptualization (lead); formal analysis (lead); funding acquisition (lead); project administration (lead); resources (lead); software (lead); supervision (lead); writing – original draft (lead); writing – review and editing (lead). **Huaxian Ma:** Methodology (equal). **Ruwaida Ahmed:** Methodology (equal).

## FUNDING INFORMATION

We are grateful for funding from the Multidisciplinary Research Program (MRP) at MD Anderson Cancer Centerand the NIH (National Institutes of Health) under R21NR019532 and P30 CA016672. Funding provided by the Joe and Dorothy Dorsett Brown Foundation and the Archer Family Foundation is gratefully acknowledged. This study was supported by the Bionutrition Research Core at MD Anderson. We also acknowledge the Center for Energy Balance in Cancer Prevention and Survivorship for their support. The authors also are thankful to the Cellular Imaging Core, Department of Leukemia for their valuable support.

## CONFLICT OF INTEREST STATEMENT

The authors declare no relevant conflicts of interest.

## ETHICS STATEMENT

Institutional Review Board Statement: The human research institutional review board (IRB) of the M.D. Anderson Cancer Center approved the study protocol 2010‐0654 (PI: Joya Chandra). For animal work, all experiments were conducted upon approval by the University of Texas MD Anderson Institutional Animal Care and Use Committee (IACUC) (IACUC 00000638 (PI: Joya Chandra)).

## INFORMED CONSENT STATEMENT

Patient consent/assent was obtained prior to data collection as approved under protocol 2010‐0654 (PI: Joya Chandra) by the University of Texas MD Anderson Cancer Center Institutional Review Board.

## Data Availability

Data sharing not applicable to this article as no datasets were generated or analyzed during the current study.
